# Predatory behaviour and taphonomy of a Jurassic belemnoid coleoid (Diplobelida, Cephalopoda)

**DOI:** 10.1038/s41598-019-44260-w

**Published:** 2019-05-28

**Authors:** Dominique Jenny, Dirk Fuchs, Alexander I. Arkhipkin, Rolf B. Hauff, Barbara Fritschi, Christian Klug

**Affiliations:** 1 Palaeontological Institute and Museum, University of Zurich, Karl Schmid-Strasse 4, 8006 Zurich, Switzerland; 2 SNSB-Bayerische Staatssammlung fuür Palaäontologie und Geologie, Richard-Wagner-Str. 10, 80333 Munich, Germany; 3 Falkland Islands Fisheries Department, FIPASS, Stanley, FIQQ 1ZZ Falkland Islands; 4 Urweltmuseum Hauff, Aichelberger Straβe 90, 73271 Holzmaden, Germany; 5 Bankstrasse 30, 8750 Glarus, Switzerland

**Keywords:** Palaeontology, Marine biology

## Abstract

We describe four complete specimens of the early squid-like cephalopod *Clarkeiteuthis conocauda* from the Toarcian Posidonienschiefer (Jurassic) each preserved with the bony fish *Leptolepis bronni* in its arms. Based on the arrangement of prey and predator, we suggest that the cephalopods caught and killed the fishes while still in well-oxygenated waters and then descended into oxygen-depleted water layers (distraction sinking) where the cephalopod suffocated. This explains the exceptional preservation, for which the Posidonienschiefer is famed. This association raises the question for the hunting behaviour of belemnoid Coleoidea. Using the proportions of soft and skeletal body parts of diplobelids and belemnitids, we estimated their body mass and buoyancy and determined the centres of mass and buoyancy. These two points were very close to each other in belemnitids, implying a low hydrodynamic stability (when ignoring the fins), while in diplobelids, the distance between those centres was greater. This suggests that diplobelids usually assumed an oblique to vertical orientation of the body axis while belemnitids could effortlessly achieve a horizontal orientation of their body. Presuming larger fins were attached to the bigger belemnitid rostra, belemnitids were better swimmers and perhaps pursuit predators while diplobelids rather ambushed their prey.

## Introduction

Among Mesozoic coleoids (cephalopods with internal hard parts that include octopuses, squid, and cuttlefish), belemnoids represent the most abundant and best documented clade. In the Jurassic, it is mainly the Belemntitida that can regionally be found in rock-forming numbers, but remains of the Diplobelida also occur occasionally. Despite their abundance, direct evidence for the swimming and hunting behaviour of these extinct cephalopods is extremely rare^1–4^.

**Figure 1:**
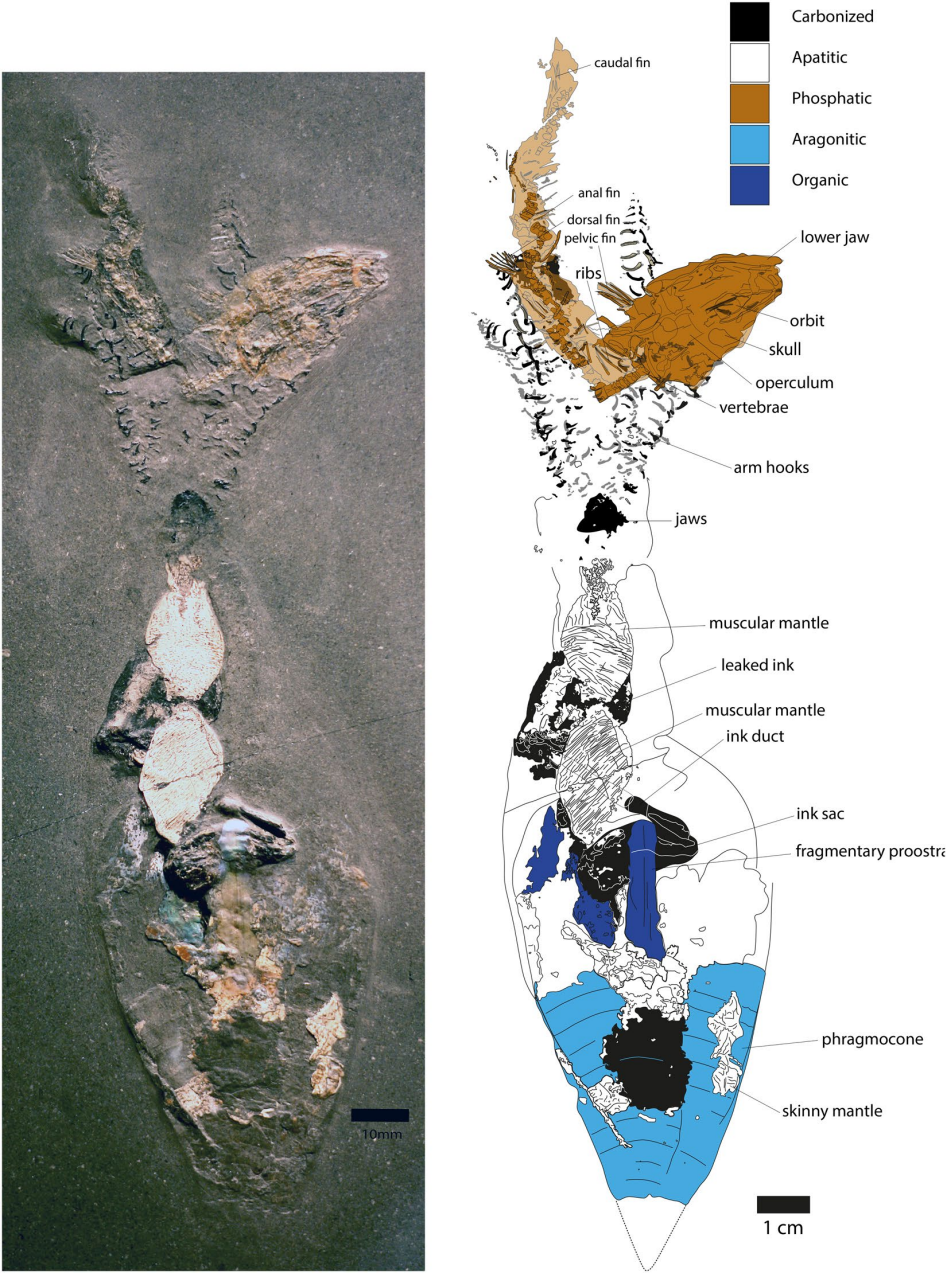
Left: Photo *Clarkeiteuthis conocauda* with *Leptolepis bronni* in its arm crown, as displayed at the Urweltmuseum in Holzmaden, Germany, Toarcian, Kirschmann Quarry. Right: Drawing of the same specimen, combining drawings of slab and counterslab.

Here, we document four cases of Early Jurassic coleoids, which all hold a small bony fish in their arm crowns (Fig. 1, Suppl. Figs 2, 4). Such preservation requires exceptional taphonomic conditions, for which the Early Jurassic Posidonienschiefer is world-renowned^5–10^. Repeated hypoxic to anoxic bottom water conditions^11,12^ decelerated decay processes and allowed pyritization or phosphatization of soft tissues that are only rarely fossilized otherwise^13–16^. These conditions persisted over a long time and in a rather vast area from southern France via northern Switzerland throughout much of Germany and into Great Britain; therefore, these widely distributed conditions allowed the formation of one of the most famous Konservatlagerstätten^8,17^, the Posidonienschiefer ( = Posidonia Shale or ‘Schistes cartons’).

From this point of view, it is not surprising that the first rostrum-bearing belemnite preserved with soft-parts was found in the Posidonienschiefer^6,18,19^. Some other cephalopods also show anatomical details of soft tissues that are usually not preserved (e.g., digestive tract in ammonites^6^; musculature and gills in non-belemoid coleoids^20–22^). Despite the extreme scarcity of cephalopods preserved with their prey, the specimen presented here was never described in detail and only figured once without detailed discussion^23^. Recently, a second specimen documenting the same behaviour was published^10^.

In the Posidonienschiefer, molluscs represent the most common macrofossils; among those, bivalves are very abundant followed by ammonites and belemnites^5,6^. Other molluscs such as non-belemnoid coleoids and nautilids are rarer and complete specimens preserved with intact arm crowns are very rare. The diplobelids described here have strong arm-hooks and a proostracum and thus belongs to the proostracum-bearing belemnoids (in contrast to, e.g., aulacoceratid belemnoids^24^). Belemnoid coleoids share a conch with mineralized shell layers including the calcitic, aragonitic or bimineralic rostrum^25–30^, which surrounds posterior parts of the chambered, cone-shaped phragmocone (aragonitic). It has been hypothesized that diplobelids could achieve neutral buoyancy using their gas-filled phragmocone^31^.

**Figure 2:**
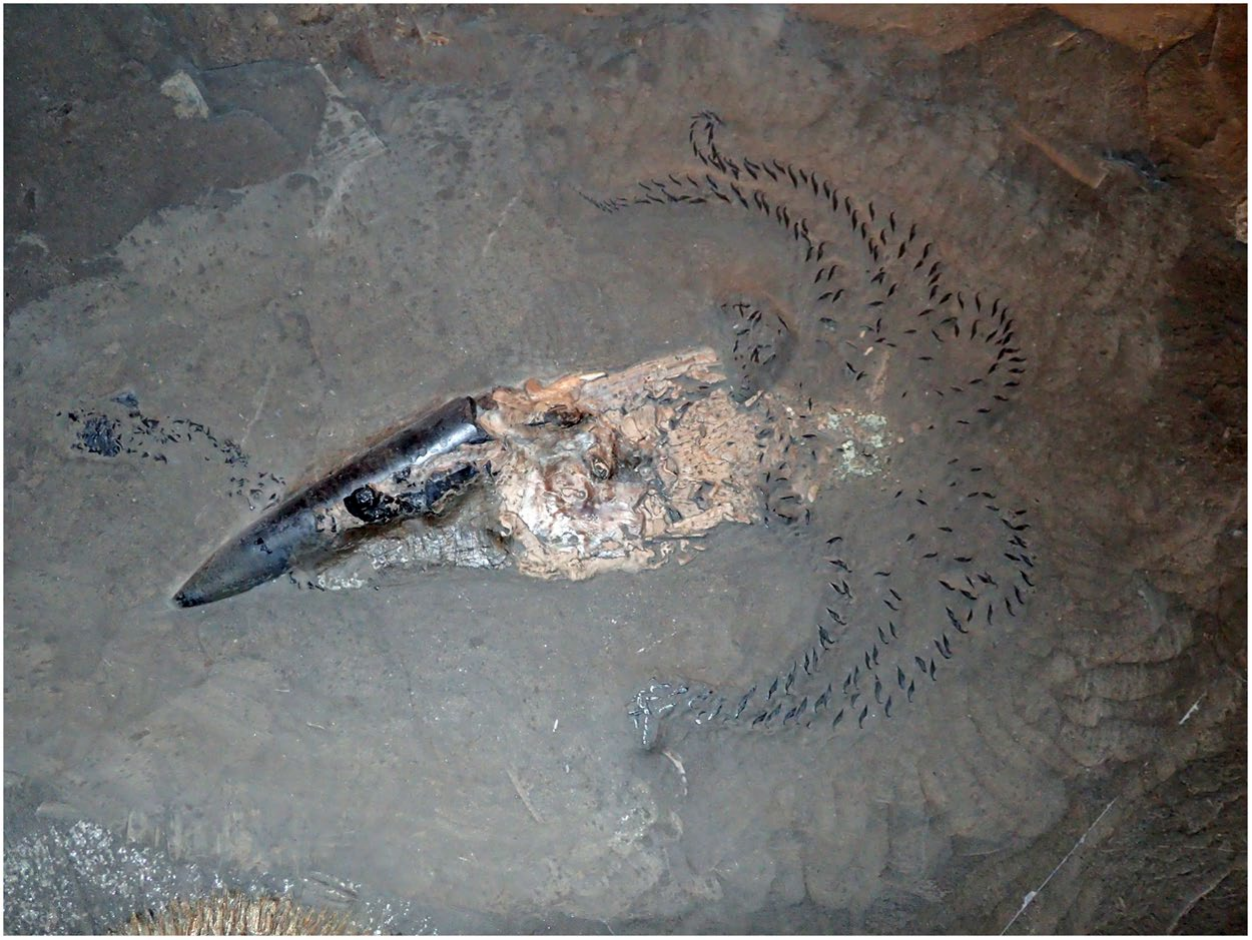
*Passaloteuthis bisulcata*, as displayed at the Urweltmuseum in Holzmaden, Germany, Toarcian. The specimen is complete preserving its rostrum, phragmocone (partially ontop of the rostrum), proostracum, phosphatized soft-tissue remains, arms with hooklets and the ink sac at the side of the rostrum. Its somewhat upended appearance can be explained by its taphonomical history, where the carcass sank into the unconsolidated sediment with the rostrum first and the other parts obliquely above it. This corroborates that the rostrum density was high during life already.

In many respects, the mode of life of belemnoid coleoids is still poorly understood but the differences in anatomy, proportions and spatial distribution of masses of the soft body and internal structures suggest diverse modes of life and habits^32^. Accordingly, we use the opportunity of the availability of these exceptionally preserved specimens of *Clarkeiteuthis conocauda* to (1) describe these specimens with their prey, (2) to discuss their taphonomic history and (3) implications for its feeding behaviour. Stimulated by this finding, we further assess (4) differences in the mode of life of *C. conocauda* and the Jurassic belemnitids *Passaloteuthis bisulcata* and *Hibolithes semisulcatus* (Belemnitida; Fig. 2, Suppl. Figs 3, 4) by reconstructing the centres of mass and buoyancy in order to make inferences on their *syn vivo* orientation in the water column.

## Description

Four specimens of the coleoid *Clarkeiteuthis conocauda* with the teleost fish *Leptolepis bronni* in their arm crown were available for study, but only one of those is kept in a public museum (Museum Hauff, Holzmaden, Germany) and thus is chosen here for the detailed description. The *C. conocauda* has a total length of 210 mm. Together with the teleost fish *L. bronni*, the two animals from the Posidonienschiefer of Holzmaden have a total length of 240 mm including arms (Fig. 1, Suppl. Fig. 7). The total length of coleoids varied during life, because they could stretch and contract the tentacles to some extent; we provide this value because we cannot directly measure the actual mantle length.

### Clarkeiteuthis conocauda

#### Phragmocone

The aragonitic phragmocone is around 64 mm long, but only 44 mm of it is preserved since the apex of the phragmocone is missing. Twelve chambers are visible, each between 4 and 5 mm wide. The last chamber measures around 40 mm in diameter. The apical angle of the crushed and flattened phragmocone is 50°, which suggests an originally lower angle prior to compaction of 30–35°^21^. The middle part of the phragmocone is covered by phosphatized remains of the skinny mantle as well as unidentified black remains.

#### Proostracum

The originally chitinous proostracum is only partially fossilized, 90 mm long and 5–9 mm wide. Taking the proportions of the animal into account, the proostracum should be 80 up to 110 mm long^21^. Possibly, only the thicker posterior parts are preserved either due to taphonomic alteration or loss during preparation. Its remains are straight and spatulate, have a mother of pearl-like shine, are slightly translucent and show straight lines along its length. It emerges from the last phragmocone chamber and reaches the top of the ink sac (posterior part of soft body). There is no evidence of a 3-lobed proostracum typical for phragmoteuthid belemnoids, thus confirming the diplobelid affinity^21^.

#### Mantle remains

*Clarkeiteuthis conocauda* shows two different types of preservation of mantle remains. Just anterior to the proostracum, two patches of the muscular mantle are visible. They have an oval shape and show the transverse striation, which is characteristic for Mesozoic coleoids and thus also for those of the Posidonienschiefer^6,19,21^. Both patches are 26 mm long and 14 mm wide and end just posterior of the jaws. Three patches of thin mantle remains lie on top of the phragmocone. Their striation is less distinct than that of the two patches mentioned first. Two of the posterior patches lie on the sides of the phragmocone and the last one covers a part of the proostracum. Additionally, a black patch lies in the middle of the phragmocone, which likely also represents mantle material^33^, perhaps pyritized in this case.

#### Ink sac and duct

The ink sac lies in the middle of the specimen and is partly covered by the proostracum. It has an oval form and is c. 23 mm long and 10 mm wide. From its right border, the 3 mm wide ink duct emerges anterolaterally to the right, then bends to the left and crosses the middle of the specimen for 14 mm. The ink duct is partially covered by mantle remains over 13 mm (dotted line in Fig. 1). A black patch that measures 23 × 20 mm lies at the anterior end of the ink duct; it is partly covered by muscular mantle and might represent leaked ink remains.

#### Arm hooks

Around 70 arm hooks are visible on the slab and 60 on the counterslab (many are probably identical, i.e. represent fragments of the same hook). Some of the hooks are arranged in rows of two, roughly documenting the dimensions of the arms and the number of arm hooks per arm (≈30, i.e. ca. 300 in total)^34,35^. The size of the arm hooks is diverse with smaller hooks at the distal ends and proximal bases of the arms and larger hooks in the middle. Their lengths vary from 1 to 5 mm with a base 0.1 to 1 mm wide. At least two different types of micro-onychites are present: Larger hooks (up to 4 mm long), where the uncinus is strongly bent as well as 1 to 5 mm long onychites, where the uncinus is just slightly curved and the shaft is straight^21^. Their black colour and structure suggest that the hooks are carbonized similar to other coleoids from the Posidonienschiefer^35^.

#### Jaws

The carbonized jaw remains are crescent-shaped and located directly posterior of the arms where the head used to be. They are c. 10 mm wide and 8 mm high. Their preservation is poor and prohibits a detailed interpretation of jaw morphology; anteriorly, however, there is a V-shaped darker structure, which likely is a part of the more strongly sclerotized part of the outer and inner lamella, possibly of the lower jaw^34,36^. The fact that this structure shows two tips points at the possibility that the jaws are still *in situ*, and one tip belongs to the lower jaw and the other to the upper jaw.

### Leptolepis bronni

The teleost fish is about 120 mm long (when straightened out), flattened and partially fragmented. Its head measures around 34 mm in length and 20 mm in height in its compacted state. The head thus makes up almost 30% of the body length. In the head region, the orbit, jaw and operculum are discernible as well as seven rays from the pectoral fin. Just posterior to the head, eight vertebrae can be seen accompanied by six ribs. The vertebral column shows several fractures and kinks, most of which probably have post mortem-causes (taphonomic) such as compaction. The vertebral column continues after a gap of around 5 mm. The course of the deformed vertebral column is well visible caudally. Remarkably, the parts on both sides of this gap each follow roughly the directions of arms adjacent to it. The most distinct kink in the vertebral column occurs exactly in this gap, and this kink is the closest to the jaw of the diplobelid.

Although the vertebral column is visible, the total number of vertebrae cannot be distinguished due to the strong compactional deformation of the specimen. Both dorsal and anal fin rays are visible near the vertebral column, with fin ray length between 1 and 5 mm. In case of the dorsal fin, the length of the distal rays is smaller than that of the dorsal ones.

### Fossilized behaviour

As mentioned above, four specimens of *Clarkeiteuthis conocauda* from Holzmaden were examined, which are all completely preserved including soft-tissue remains. All four hold a small teleost fish in their arm crowns. To our knowledge, these represent – apart from an octobrachian (*Glyphiteuthis libanotica*) from the Upper Cretaceous Lebanon Limestones^37^ – the first and oldest findings of a cephalopod that has been preserved together with its vertebrate prey within its arm crown (stomach contents with vertebrate remains have occasionally been reported from fossil coleoids^1,37–40^ and once prey within the buccal mass^41^). The association of small teleosts within arm crowns of *C. conocauda* can be considered as evidence for fossilized predatory behaviour, because both species are rather rarely preserved in articulation otherwise, and thus, an accidental association of such fossils is unlikely; more importantly, the same joint occurrence in always the same way in four cases corroborates the interpretation as fossilized behaviour. If currents had transported the fish to the coleoid carcass, the arms would have been bent in current direction and the fish would probably have come to a rest somewhere else next to the diplobelid with the long body axes of both animals aligned in parallel.

The interpretation of the fish as the last prey is further corroborated by behavioural biology studies of recent squids. For example, *Illex illecebrosus* captures its prey with both head and tail protruding from the squid’s arms^42^. To immobilize the prey, squids cut the vertebral column of their fish prey by a jaw bite near the head^43^. In three of the four cases, the fossil fish displays a distinct kink in vertebral column in close proximity to the beak of the diplobelid, thereby documenting this behaviour for *Clarkeiteuthis*.

A possible explanation as to how these specimens became preserved is the phenomenon of post capture sinking or rising, which has been reported from, e.g., the giant squid *Architeuthis dux*^44,45^ and from *Illex illecebrosus*^43^. Some pelagic animals are slightly negatively buoyant and start sinking when inactive. *Architeuthis* has ammonium chloride-solution-filled tissues that neutralize buoyancy^46^, but if its prey is negatively buoyant or tries to escape downward, the squid gets pulled downward as well. Consequently, if *Clarkeiteuthis* focused on its prey instead of staying in the same water level, they likely began to sink, or, alternatively, the cephalopod descended on purpose in order to get out of sight of other predators. This downward movement was potentially accelerated by the reduction of the gas volume in the teleost’s swim bladder by the increasing hydrostatic pressure. Thereby, the living coleoids with the probably already dead fish reached the poorly oxygenated bottom waters and suffocated, which is supported by the ongoing embrace of the fish by the diplobelid in all four cases. Post capture sinking is not only corroborated by the completeness of the cephalopod and its prey but also by the fact that sinking can still be seen in recent cephalopod species as well as in other fossilized specimens like mating lacustrine turtles^47^. Recently, Mapes *et al*.^48^ coined the term ‘distraction sinking’ for aquatic organisms that were occupied by activities such as mating, feeding, or fighting; this activity distracted their attention from the onset of a sinking process, whereby they potentially reached deeper water layers poor in oxygen (as in the case of the *Clarkeiteuthis* described here) or poisonous (as in the case described by Joyce *et al*.^47^).

**Figure 3:**
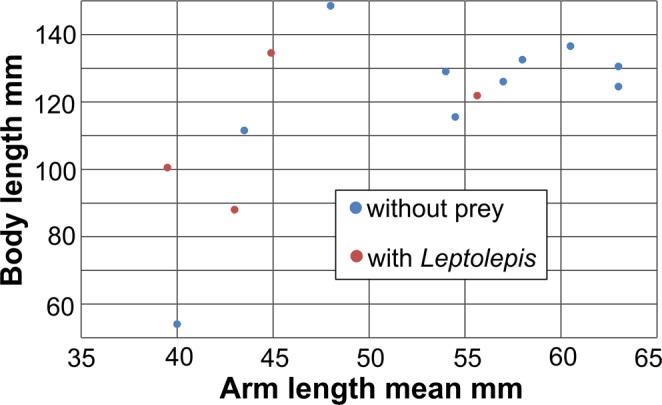
Relative arm lengths in the four specimens of *Clarkeiteuthis conocauda* with *Leptolepis bronni* in its arm crown and ten specimens of Jurassic diplobelids without prey. Note that the specimens with fish tend to have rather contracted arms.

Cephalopods usually pull prey towards them by contracting their arms or tentacles^49–51^. Did *Clarkeiteuthis* also contract its arms after it had caught the fish? We measured the lengths of the visible arms of 14 specimens and calculated their mean length (Fig. 3). As a proxy for body size we used the distance between the most proximal arm hook and the last formed septum. With measurements of only 14 specimens with only four with a fish in their arm crown, the statistical power is poor. Additionally, arms can be contracted for several reasons besides holding prey; it might occur due to distress, linked with swimming movements or else^52,53^. In Fig. 3, mean arm length is plotted versus body size. The four specimens holding a fish are among the specimens with the most contracted arms. There is only one specimen with similarly contracted arms, but the reason for the contraction is not evident. It is possible, that the prey is not preserved, not visible or escaped before the squid settled on the sediment but there might be other reasons to explain the contracted arms of this specimen (Nr. 2 in Table 1). In any case, these findings suggest that *Clarkeiteuthis* indeed contracted its arms to pull its teleost prey towards its mouth to cut the spine and then feed on it.

**Table 1:** Lengths of arms (arm numbering is arbitrary), phragmocone and body of published and museum specimens of *Clarkeiteuthis*.

	Species	source	fish	arm 1	arm 2	arm 3	arm 4	arm 5	arm 6	arm 7	arm 8	arm 9	arm 10	mean	phragmocone length	total length	size
1	*cono-cauda*	Museum Hauff, arm crown lateral	no	61.3	61.3	51.3	50	50	48.75	50	56.3	58.8		54.2	56.3	238.8	128.3
2	*cono-cauda*	Museum Hauff, arms spread	no	38.3	37.5	41.7	43.3	47.5	39.2	38.3	41.7	40.8	33.3	40.2	65	159.2	54
3	*cono-cauda*	this paper	yes	38.5	43.1	51.6	39.3	43.1						43.1	57.8	188.7	87.8
4	*cono-cauda*	Col. Klaschka^10^	yes	44.4	50	29.4	37.8	41.1	47.8	25.6				39.4	60	200	100.6
5	*cono-cauda*	Hauff & Hauff ^5^: p. 101	no	64	64	65.6	57.6	60.8	64	60.8	68.8			63.2	59.2	246.4	124
6	*monte-fiorei*	Hart *et al*.^67^	no	61.5	49.4	42.2	42.2	44.6						48	59	255.5	148.5
7	*monte-fiorei*	Hart *et al*.^67^	no	45.9	44.9	40.8	40.8	44.9						43.5	52	207.1	111.6
8	*cono-cauda*	Fuchs *et al*.^21^, Museum Hauff 5212	no	40.9	61.8	61.8	57.3	59.1	60	60.9	68.2	40.9		56.8	70	25.27	126
9	*cono-cauda*	Fuchs *et al*.^21^, Museum Hauff 5205	no	47.3	50	59.1	60	55.4						54.4	50.9	220.9	115.6
10	*cono-cauda*	Fuchs *et al*.^21^, Museum Hauff 5225	no	60	57.9	62.1	51.6							57.9	78.9	269.5	132.6
11	*cono-cauda*	Fuchs *et al*.^21^, Ohmden 1341	no	66.4	58.2	68.2	66.4	62.7	59.1	58.2				62.7	65.5	259.1	130.9
12	*cono-cauda*	Fuchs *et al*.^21^, Museum Hauff 5216	no	55.5	65.5	58.2	59.1	68.2	61.8	54.5				60.4	50	247.3	136.9
13	*cono-cauda*	Col. Weber	yes	44	43	43	42	56	45					45.5	67	244	131.5
14	*cono-cauda*	Col. Weber	yes	48	50	53	65	64						56	57	235	122

Remarkably, only one type of prey has been found in the arm crown of *Clarkeiteuthis* to our knowledge. The specimen presented here, the one published by Klaschka^10^ as well as the two additional specimens (Suppl. Fig. 2) all hold the small teleost *Leptolepis* in their arms. This raises the question for prey specificity. With four specimens available, the statistical power is low, but it is remarkable that, for example, no specimen with invertebrate prey is known and that it is always the same fish that has been caught. Articulated fish skeletons are rare in the Posidonienschiefer, but among all fishes, *Leptolepis* is one of the more common genera. Being one of the smallest, it was one of the very few species *Clarkeiteuthis* could probably handle without the risk of becoming injured or killed. In contrast to other small contemporary fish such as *Tetragonolepis*, *Leptolepis* lacked thick scales. With this reduced armour, it was probably an easier prey to catch and hold for the diplobelids by inserting their hooks in the skin and flesh rather than into those species with thick scales. Late Jurassic relatives (*Leptolepides*) of the Toarcian *Leptolepis* occur in large fossilized groups suggesting that they often lived in larger schools^54^. Thus, *Leptolepis* was possibly quite abundant and a likely prey but their delicate skeletons became quickly disarticulated. In any case, these joint occurrences suggest an overlapping habitat in the water column of both cephalopod and teleost.

## Mode of Life

**Figure 4:**
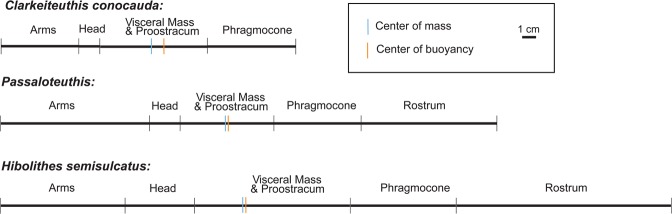
Localizations of the centres of mass (blue cross) and buoyancy (black cross) in *Clarkeiteuthis conocauda, Passaloteuthis bisulcata* and *Hibolithes semisulcatus* along the body axis in relation to their body parts.

In order to reconstruct the syn vivo body orientation and the mode of life of *Clarkeiteuthis conocauda*, different approaches were employed. The first approach included the determination of the centres of buoyancy and mass of *Clarkeiteuthis conocauda, Passaloteuthis bisulcata* and *Hibolithes semisulcatus* (these taxa were chosen because of the well known phragmocone and rostrum proportions). In *Clarkeiteuthis*, the centre of buoyancy lies more or less in the centre of its body, around 32 mm anterior to the edge of the last septum of the phragmocone (Fig. 4, Suppl. Fig. 1). The specimen had its centre of mass in the middle of its body, about 40–41 mm anterior to the phragmocone, which is very close to its centre of buoyancy with around 8–9 mm separating them. The centre of buoyancy of *Hibolithes* lied around 66 mm anterior to the phragmocone (Fig. 4). In *Hibolithes*, the centre of mass was situated in the middle of the body, about 68 mm anterior to the phragmocone. This implies that its centres of mass and buoyancy were only about 2 mm apart.

In *Passaloteuthis bisulcata*, the centre of buoyancy lied 33 mm anterior to the phragmocone, whereas the centre of mass lied 35 mm anterior to it. As in *Hibolithes*, the centres of mass and buoyancy were situated around 2 mm apart from each other.

These values vary depending on the values used for the mass of the phragmocone and rostrum density. If the phragmocone was entirely filled with gas (which implies estimated phragmocone weights of 2.32 g for *Clarkeiteuthis*, 1.68 g for *Passaloteuthis* and 1.49 g for *Hibolithes*), the centre of mass lay closer to the head. If the phragmocone was filled with 30% liquid and 70% gas (which implies estimated weights of 13.49 g for *Clarkeiteuthis*, 7.99 g for *Passaloteuthis* and 10.99 g for *Hibolithes*), the centre of mass shifted backwards^55^. With a centre of mass in the middle of the body and close to the centre of buoyancy, the animal would have been able to turn its body into a horizontal position but it could rather easily turn in every direction. Accordingly, the animal presumably could maintain a stable horizontal position using its fins.

Mean body densities of *Clarkeiteuthis, Passaloteuthis* and *Hibolithes* were calculated to verify the calculations of their modes of life of using the values listed in Table 2. At a total length (including arms) of approximately 213 mm, *Clarkeiteuthis* weighed around 145.8 to 146.6 g and its body had a volume of 161.6 to 162.2 cm^3^ which implies a mean density of 0.90 up to 0.904 g/cm^3^. At a total length of approximately 300 mm (including arms), *Passaloteuthis* weighed around 170.9 to 179.3 g, had a body volume of 175.2 to 175.7 cm^3^ and thus a mean density between 1.004 and 1.051 g/cm^3^. At a total length (including arms) of about 456 mm, *Hibolithes* weighed around 547.8 to 560.5 g, had a body volume of 539.7 to 541.4 cm^3^ and a mean density of 1.011 to 1.035 g/cm^3^. Compared to the density of seawater of 1.025 g/cm^3^ at 3.5% salinity, *Hibolithes* would have been nearly neutrally buoyant with a phragmocone filling of about 12 to 53% of its volume; similarly, *Passaloteuthis* would have been neutrally buoyant with a phragomocone filling of around 33 to 73% and the diplobelid would have been positively buoyant up to a phragmocone filling of nearly 82 to 86%.

**Table 2:** The table shows reconstructions of volumes and densities of *Passaloteuthis bisulcata*, *Hibolithes semisulcatus*, and *Clarkeiteuthis conocauda*, which were used for the calculations of the centre of mass and buoyancy of this species^29,30^.

Bodypart	Material	Density (g/cm^3^)	Volume (cm^3^)	Weight (g)
**Passaloteuthis**				
Arms	Organic	1.055	27.500	29.013
Head	Organic	1.055	113.730	119.985
Muscular mantle	Organic	1.055	349.109	368.310
Proostracum min.	Chitinous	1.48	0.407	0.603
Proostracum max.	Chitinous	1.48	2.036	3.014
Phragmocone total volume			31.835	
Phragmocone hard parts (0% liquid)	Aragonitic	2.620	0.856	2.243
Liquid inside phragmocone 30%	Cameral liquid	1.013	9.294	9.414
Phragmocone filled 30%	Aragonitic/cameral liquid	1.013/2.620	10.150	11.658
Rostrum min.	Calcite	1.100	6.239	6.863
Rostrum max.	Calcite	1.700	6.239	10.607
**Hibolithes**				
Arms	Organic	1.055	24.020	25.341
Head	Organic	1.055	7.940	8.377
Muscular mantle	Organic	1.055	108.856	114.843
Proostracum min.	Chitinous	1.48	0.126	0.186
Proostracum max.	Chitinous	1.48	0.629	0.931
Phragmocone total volume			21.375	
Phragmocone hard parts (0% liquid)	Aragonitic	2.620	0.000	0.000
Liquid inside phragmocone 30%	Cameral liquid	1.013	6.413	6.496
Phragmocone filled 30%	Aragonitic/cameral liquid	1.013/2.620	6.413	6.496
Rostrum min.	Calcite	1.100	12.850	14.135
Rostrum max.	Calcite	1.700	12.850	21.845
Rostrum max.	Calcite	1.700	6.239	10.607
**Clarkeiteuthis**				
Arms	Organic	1.055	8.629	9.103
Head	Organic	1.055	10.011	10.561
Muscular mantle	Organic	1.055	104.364	110.104
Proostracum min.	Chitinous	1.480	0.034	0.050
Proostracum max.	Chitinous	1.480	0.579	0.856
Phragmocone total volume			37.656	
Phragmocone hard parts (0% liquid)	Aragonitic	2.620	0.000	0.000
Liquid inside phragmocone 30%	Cameral liquid	1.013	11.297	11.444
Phragmocone filled 30%	Aragonitic/cameral liquid	1.013/2.620	11.297	11.444
Rostrum		0.00	0.000	0.000

The second approach to reconstruct the mode of life for diplobelids and belemnitids are actualistic comparisons with recent cephalopods such as cranchiids and other teuthids. Due to their comparatively thin muscular mantle, the modern cranchiids are buoyant but poor swimmers, which often position themselves with the head down in the water column^56,57^. By contrast, modern non-buoyant teuthids (e.g. loliginids, ommastrephids or onychoteuthids) are mostly very muscular and can migrate over long distances. Belemnitids might have had a similar mode of life due to their anatomical features.

The Cranchiidae vary in mantle length between 100 and 2000 mm^57,58^. The sometimes translucent animals have an elongated, conical or cylindrical shape, a thin but muscular mantle and a short head with large eyes^59^. Their fins vary in size and shape depending on species and habit. The thin fins are either widely separate, small and paddle-shaped or medium to large and round^60^. Cranchiids are neutrally buoyant due to large coelomic cavities, which are filled with an ammonium chloride-solution (NH_4_Cl^59,61^). They are able to change their orientation in the water column by using their fins rather than by contracting the mantle cavity, i.e. hyponome action^61^. Due to these features, cranchiids are not very active swimmers but position themselves with the head down in the water column. In that respect, the diplobelids might have had a similar mode of life due to their supposed *syn vivo* position.

## Discussion

Reconstructing the mode of life of Jurassic coleoids is of interest because they are quite common in many localities and because the rostrum of belemnitids is widely used for examinations of stable isotope ratios to measure palaeotemperatures^62,63^. Such isotope data recently turned out to be far from trivial to interpret^26,63^. Other evidence for coleoid habitats and behaviour is rare; partially, this roots in the scarcity of complete specimens including soft parts, which are largely limited to Konservatlagerstätten^32,46^.

Most researchers suggest that belemnitids used their large and supposedly heavy calcitic rostra to obtain a horizontal swimming position with further support from the fins^31^. Information on the mode of life of diplobelids without a massive rostrum was missing.

According to our buoyancy calculations of *Clarkeiteuthis*, the centre of gravity lies in the middle of the animal and moderately close to the centre of buoyancy using the values listed in Table 2. This suggests that diplobelids could easily change their orientation from oblique to vertical. This also depended on the amount of chamber water in the phragmocone. If the phragmocone was partially filled with liquid, the orientation would have been rather horizontal to oblique. If the diplobelid maintained a vertical body orientation, this would imply that they were less active swimmers and thus rather planktonic with the capability of occasional short bursts of rapid swimming movements (to catch prey or to escape predators).

Belemnoteuthids such as *Acanthoteuthis* were suggested to have been fast swimmers^32^, which is partially based on a misinterpretation of the term ‘buoyant squid’. In contrast to Klug *et al*.^32^, *Acanthoteuthis* was probably rather nektoplanktonic, as suggested by the shape and dimensions of its statoliths, the thin aragonitic rostrum and the probably rather thin mantle (poorly preserved compared to the thicker mantle in, e.g., *Plesioteuthis* or *Leptotheuthis*).

In belemnitids with their characteristic partially calcitic rostrum^64,65^, the centre of mass and buoyancy were very close to each other according to our reconstructions. This result suggests that belemnitids with a comparatively long rostrum such as *Passaloteuthis* and *Hibolithes* could easily turn their body in each direction and thus also horizontally. The well-developed fins^32,64^ enabled the animal to steer, implying an at least temporally nektonic mode of life. Also, accepting that the rostrum supported fins^31,65^, this suggests that larger fins were present in belemnitids than in diplobelids, thus providing more dynamic lift^31^.

The calculations of the mean density for all three cephalopods show that their phragmocones were partially filled with liquid, because only then, they attained neutral buoyancy^57^; it is unclear, however, if these cephalopods had other organs controlling their average body density, thus making them neutrally (like the more passive planktonic squids) or slightly negatively buoyant (like nektonic ommastrephids and loliginids) with less chamber liquid. For *Passaloteuthis* and *Hibolithes*, the density of 1.004 to 1.051 g/cm^3^, is close to that of seawater, which corroborates the hypothesis of neutral or near-neutral buoyancy. With a low mean density of possibly 0.9 g/cm^3^, *Clarkeiteuthis* was rather strongly positively buoyant (which is unlikely) and thus, a higher amount of chamber liquid in the phragmocone is quite likely. These differences in density resulted from the small phragmocone and large rostrum of *Passaloteuthis* and *Hibolithes* in comparison to *Clarkeiteuthis* with a large phragmocone but a small sheath-like rostrum. This implies that both taxa regulated buoyancy by the quantity of chamber liquid^31,46^. From its morphology (rostrum, mantle) and the spatial distribution of mass, we conclude that *Clarkeiteuthis* might have had a mode of life similar to modern neutrally buoyant squids such as the cranchiids, oblique to vertical orientation of the body axis, a life as ambush predator (arm hooks, teleost prey) that stayed inactive most of the time but was capable of short pulses of fast swimming to catch prey.

If diplobelids were neutrally buoyant, it would have been possible that they changed their orientation in the water column similar to modern cranchiids by the use of their fins and funnel in addition to contracting their thin muscular mantle^66^. Assuming the presence of rather well-developed fins (concluded from rostrum size^32^) and a thick muscular mantle, belemnites were probably capable of using the fins and mantle contractions for more enduring and rapid swimming for, e.g., active pursuit hunting. Taking the even thicker muscular mantle in other Jurassic coleoids such as *Plesioteuthis*, *Trachyteuthis* or *Leptotheuthis* into account, these gladius-bearing octobrachians were likely the best swimmers among the Jurassic coleoids.

## Conclusions

**Figure 5:**
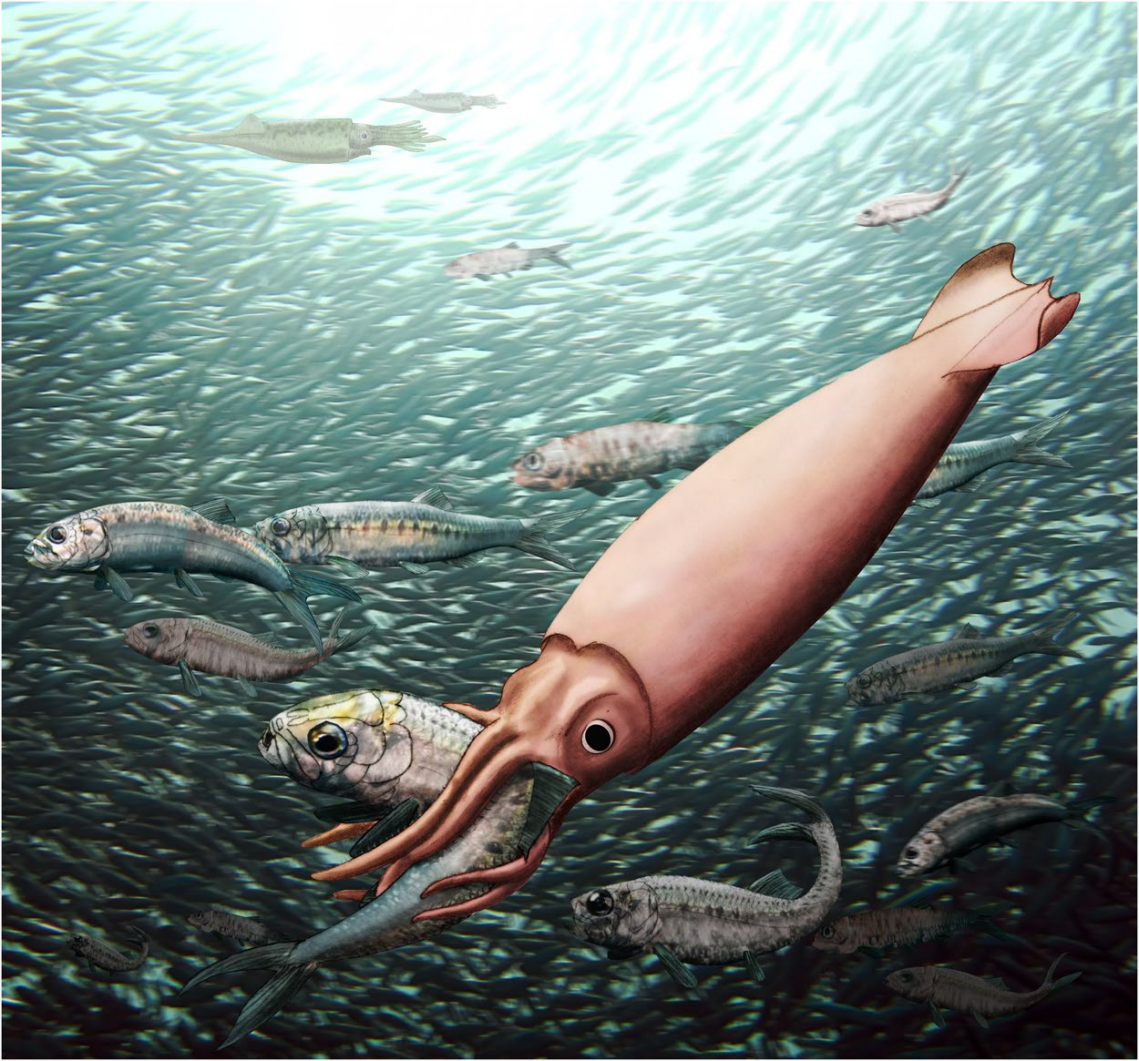
Reconstruction of *Clarkeiteuthis conocauda* after catching the small teleost *Leptolepis bronni*. Note the horizontally swimming individuals of *Passaloteuthis bisulcata* near the water surface. Artwork by CK.

We have described adult individuals of *Clarkeiteuthis conocauda*, which caught small teleost fish of the species *Leptolepis bronni*. This diplobelid cephalopod was able to catch and hold on to the fish with its numerous arm hooks (Fig. 5). Having caught the fish, it likely first contracted its arms to bring the fish towards its mouth. Like some modern cephalopods, these diplobelids probably quickly killed the fish by cutting its spine, which is documented in distinct kinks in the vertebral column in three of the four specimens. The kink in the spine lies the closest to the cephalopod’s mouth. Once the teleost prey was immobilized, both animals started to sink, perhaps due to the deflated swim bladder of the fish, because the cephalopod tried to move out of sight of other predators or it stopped active movements. Both animals sunk into the oxygen depleted bottom waters where the cephalopod suffocated (the low oxygen conditions explain the exceptional preservation). These specimens provide therefore evidence for their predatory behaviour and indirect evidence for post capture sinking, which is a special case of distraction sinking^47^. Thus, diplobelids played an ecological role as neutrally buoyant medium-sized ambush predators in Jurassic food webs. The fact that all four specimens of *Clarkeiteuthis* were fossilized with a *Leptolepis* in their arm crown suggests that there possibly was some sort of prey specificity. Further indirect evidence for the hunting behaviour comes from their body orientation in the water during life; reconstructions of the centres of mass and buoyancy suggest a possible vertical orientation for diplobelids in contrast to a preferentially horizontal orientation of belemnitids.

## Supplementary information

Supplementary information

## References

[1] Keupp H, Engeser T, Fuchs D, Haechel W (2010). Ein *Trachyteuthis hastiformis* (Cephalopoda, Coleoidea) mit Spermatophoren aus dem Ober-Kimmeridgium von Painten (Ostbayern). Archaeopteryx.

[2] Keupp H, Saad PA, Schweigert G (2016). Nautiliden mit Kiefern und Mageninhalt. Fossilien.

[3] Kruta I, Landman NH, Rouget I, Cecca F, Larson NL (2010). The Jaw apparatus of the Late Cretaceous ammonite. Didymoceras. J. Paleontol..

[4] Klug, C. & Lehmann, J. Soft part anatomy of ammonoids: Reconstructing the animal based on exceptionally preserved specimens and actualistic comparisons. In: Klug, C., Korn, D., De Baets, K., Kruta, I. & Mapes, R. H. (eds). *Ammonoid paleobiology: From anatomy to ecology*. Springer, Dordrecht, 507–529 (2015).

[5] Hauff, B. & Hauff, R. B. *Das Holzmadenbuch*. Museum Hauff, Holzmaden ⁄ Teck (1981).

[6] Riegraf, W., Werner, G. & Lörcher, F. *Der Posidonienschiefer. Biostratigraphie, Fauna und Fazies des südwestdeutschen Untertoarciums (Lias epsilon)*. Enke, Stuttgart (1984).

[7] Urlichs M, Wild R, Ziegler B (1994). Der Posidonien-Schiefer und seine Fossilien. Stuttg. Beitr. Naturkd..

[8] Bottjer, D. J., Etter, W., Hagadorn, J. W. & Tang, C. M. Fossil-Lagerstätten: Jewels of the Fossil Record. In *Exceptional Fossil Preservation*, Columbia University Press, New York (2002).

[9] Etter, W. & Tang, C. M. Posidonia shale: Germany’s Jurassic marine park. In Bottjer, D. J., Etter, W., Hagadorn, J. W. & Tang, C.: *Exceptional Fossil Preservation*. Columbia University Press, New York, 265–291 (2002).

[10] Klaschka J (2018). Ein taphonomischer Jackpot – Tintenfisch bei Fischmahlzeit von Ichthyosaurier erbeutet. Fossilien.

[11] Röhl HJ, Schmid-Röhl A, Oschmann W, Frimmel A, Schwark L (2001). The Posidonia Shale (Lower Toarcian) of SW-Germany: An oxygen-depleted ecosystem controlled by sea level and palaeoclimate. Palaeogeogr., Palaeoclim., Palaeoecol..

[12] Röhl A, Schmid-Röhl HJ, Oschmann W, Frimmel A, Schwark L (2002). Palaeoenvironmental reconstruction of Lower Toarcian epicontinental black shales (Posidonia Shale, SW Germany): Global versus regional control. Geobios.

[13] Allison PA (1988). Konservat-Lagerstätten: cause and classification. Paleobiology.

[14] Briggs DEG, Kear AJ, Martill DM, Wilby PR (1993). Phosphatization of soft-tissue in experiments and fossils. J. Geol. Soc., London.

[15] Kear AJ, Briggs DEG, Donovan DT (1995). Decay and fossilization of non-mineralized tissue in coleoid cephalopods. Palaeontology.

[16] Briggs DEG, Wilby PR (1996). The role of the calcium carbonate-calcium phosphate switch in the mineralization of soft- bodied fossils. J. Geol. Soc., London.

[17] Seilacher A (1970). Arbeitskonzept zur Konstruktionsmorphologie. Lethaia.

[18] Reitner J, Urlichs M (1983). Echte Weichteilbelemniten aus dem Untertoarcium (Posidonienschiefer) Südwestdeutschlands. N. Jb. Geol. Paläont..

[19] Riegraf W, Hauff R (1983). Belemniten mit Weichkörper, Fangarmen und Gladius aus dem Untertoarcium (Posidonienschiefer) und Unteraalenium (Opalinuston) Südwestdeutschlands. N. Jb. Geol. Paläont..

[20] Reitner J, Mehl J (1989). Ein besonderes Fossil. Paläontol. Z..

[21] Fuchs D, Donovan DT, Keupp H (2013). Taxonomic revision of „*Onychoteuthis*“ *conocauda* Quenstedt, 1849 (Cephalopoda: Coleoidea). N. Jb. Geol. Paläont. Abh..

[22] Donovan DT, Fuchs D, Part M (2016). Chapter 10: Fossilized soft tissues in Coleoidea. Treatise Online.

[23] Seilacher, A. & Gishlick, A. D. *Morphodynamics*. CRC Press, Taylor & Francis Group, Boca Raton (2015).

[24] Engeser T (1990). Phylogeny of the fossil coleoid Cepalopoda (Mullusca). Berl. Geowiss. Abh..

[25] Fuchs D (2012). The “rostrum”-problem in coleoid terminology-an attempt to clarify inconsistencies. Geobios.

[26] Hoffmann R (2016). Evidence for a composite organic-inorganic fabric of belemnite rostra: Implications for palaeoceanography and palaeoecology. Sedimentary Geology.

[27] De Baets K, Munnecke A (2018). Evidence for Palaeozoic orthoconic cephalopods with bimineralic shells. Palaeontology.

[28] Linzmeier, B. Refining the interpretation of oxygen isotope variability in free-swimming organisms. *PaleorXiv Papers*, 20 pp., 10.31233/osf.io/nbtgm (2018; preprint).

[29] Kröger B, Vinther J, Fuchs D (2011). Cephalopod origin and evolution: A congruent picture emerging from fossils, development and molecules. Bioessays.

[30] Tanner AR (2017). Molecular clocks indicate turnover and diversification of modern coleoid cephalopods during the Mesozoic Marine Revolution. The Royal Society.

[31] Monks N, Hardwick JD, Gale AS (1996). The function of the belemnite guard. Paläont. Z..

[32] Klug C, Fuchs D, Schweigert G, Kruta I, Tischlinger H (2016). Adaptations to squid-style high-speed swimming in Jurassic belemnitids. Biology letters.

[33] Doguzhaeva LA, Summesberger H, Mutvei H, Brandstaetter F (2007). The mantle, ink sac, ink, arm hooks and soft body debris associated with the shells in Late Triassic coleoid cephalopod *Phragmoteuthis* from the Austrian Alps. Paleoworld.

[34] Klug C, Schweigert G, Fuchs D, Dietl G (2010). First record of a belemnite preserved with beaks, arms and ink sac from the Nusplingen Lithographic Limestone (Kimmeridgian, SW Germany). Lethaia.

[35] Hoffmann R, Weinkauf MFG, Fuchs D (2017). Grasping the shape of belemnoid arm hooks- a quantitative approach. Paleobiology.

[36] Nixon M, Part M (2015). Chapter 12: The buccal apparatus of Recent and fossil forms. Treatise Online.

[37] Fuchs D, Larson N (2011). Diversity, Morphology, and Phylogeny of Coleoid Cephalopods from the Upper Cretaceous Plattenkalks of Lebanon-Part II: Teudopseina. J. Paleontol..

[38] Landman NL, Davis RA (1988). Jaw and crop preserved in an orthoconic nautiloid cephalopod from the Bear Gulch Limestone (Mississippian, Montana). New Mexico Bureau of Mines and Mineral Resources.

[39] Mapes, R. H., Weller, E. A. & Doguzhaeva, L. A. Cephalopods showing a tentacle with arm hooks and an ink sac from Montana, USA. In Tanabe, K., Shigeta, Y., Sasaki, T. & Hirano, H. 2010. *Cephalopods - Present and Past*. Tokai University Press, Tokyo, 155–170 (2010).

[40] Fuchs D, Bracchi G, Weiss R (2009). New Octopods (Cephalopoda: Coleoidea) from the Late Cretaceous (upper Cenomanian) of Hâkel and Hâdjoula, Lebanon. Palaeontology.

[41] Fuchs D, Larson N (2011). Diversity, Morphology, and Phylogeny of Coleoid Cephalopods from the Upper Cretaceous Plattenkalks of Lebanon-Part I: Prototeuthidina. J. Paleontol..

[42] Foyle TP, O’dor RK (1988). Predatory strategies of squid (*Illex illecebrosus*) attacking small and large fish. Marine & Freshwater Behaviour & Phys..

[43] Rodhouse PG, Nigmatullin CM (1996). Role as consumers. Philosophical Transactions of the Royal Society B.

[44] Bolstad KS, O’Shea S (2004). Gut contents of a giant squid *Architeuthis dux* (Cephalopoda: Oegopsida) from New Zealand waters. New Zealand Journal of Zoology.

[45] Kubodera T, Mori K (2005). First-ever observations of a live giant squid in the wild. Proc. R. Soc..

[46] Clements T, Colleary C, De Baets K, Vinther J (2016). Buoyancy mechanisms limit preservation of Coleoid cephalopod soft tissues in Mesozoic Lägerstätten. Paleontology.

[47] Joyce WG, Micklich N, Schaal SFK, Scheyer TM (2012). Caught in the act: The first record of copulating fossil vertebrates. Biology letters.

[48] Mapes, R. H., Landman, N. H. & Klug, C. Caught in the act? *Swiss J. Palaeontol*. ca. 12 pp. (in press, 2019).

[49] Kier WM (1982). The functional-morphology of the musculature of squid (Loliginidae) arms and tentacles. Journal of Morphology.

[50] Kier WM (2016). The musculature of coleoid cephalopod arms and tentacles. Frontiers in Cell and Developmental Biology.

[51] van Leeuwen JL, Kier WM (1997). Functional design of tentacles in squid: linking sarcomere ultrastructure to gross morphological dynamics. Phil. Trans. Royal Soc. B.

[52] Mather JA, Griebel U, Byrne RA (2010). Squid dances: an ethogram of postures and actions of Sepioteuthis sepioidea squid with a muscular hydrostatic system. Marine and Freshwater Behaviour and Physiology.

[53] Klug C, Fuchs D, Schweigert G, Röper M, Tischlinger H (2015). New anatomical information on arms and fins from exceptionally preserved *Plesioteuthis* (Coleoidea) from the Late Jurassic of Germany. Swiss J. Palaeontol..

[54] Arratia, G., Schultze, H.-P., Tischlinger, H. & Viohl, G. *Solnhofen. Ein Fenster in die Jurazeit*. Pfeil, München (2015).

[55] Tajika A (2015). Empirical 3D model of the conch of the Middle Jurassic ammonite microconch *Normannites*: Its buoyancy, the physical effects of its mature modifications and speculations on their function. Historical Biology.

[56] Seapy RR, Young RE (1986). Concealment in epipelagic pterotracheid heteropods (Gastropoda) and cranchiid squids (Cephalopoda). J. Zool., Lond. (A).

[57] Vecchione M, Roper CFE (1991). Cephalopods observed from submersibles in the western North Atlantic. Bulletin of Marine Science.

[58] Voss NA, Stephen SJ, Dong Z (1992). Family Cranchiidae Prosch, 1849. Smithsonian Contributions to Zoology.

[59] Arkhipkin AI (1996). Statolith microstructure and age of early life stages of planktonic squids *Galiteuthis phyllura* and *Belonella borealis* (Oegopsida, Cranchiidae) from the northern North Pacific. Journal of Plankton Research.

[60] Voss NA (1980). A generic revision of the Cranchiidae (Cephalopoda; Oegopsida). Bulletin of marine science.

[61] Clarke MR (1962). Respiratory and swimming movements in the cephalopod *Cranchia scabra*. Nature.

[62] Mutterlose J, Malkoc M, Schouten S, Damsté JSS, Forster A (2010). TEX86 and stable d^18^O paleothermometry of early Cretaceous sediments: Implications for belemnite ecology and paleotemperature proxy application. Earth Planetary Sci. Lett..

[63] Li Q (2013). Evaluating Mg/Ca in belemnite calcite as a palaeo-proxy. Palaeogeogr. Palaeoclim. Palaeoecol..

[64] Naef, A. *Die fossilen Tintenfische*. Verlag von Gustav Fischer, Jena (1922).

[65] Arkhipkin A, Weis R, Mariotti N, Shcherbich Z (2015). ‘Tailed’, cephalopods. J. Moll. Stud..

[66] Clarke MR, Denton EJ, Gilpin-Brown JB (1979). On the use of ammonium for buoyancy in squids. Journal of the Marine Biological Association of the UK.

[67] Hart, M. B., Hughes, Z., Page, K. N., Price, G. D. & Smart, C. W. Arm hooks of coleoid cephalopods from the Jurassic succession of the Wessex Basin, Southern England. *Proc. Geol. Assoc*, 10.1016/j.pgeola.2018.02.008 (2018).

