# Supplementary material for: Predatory behaviour and taphonomy of a Jurassic belemnoid coleoid (Diplobelida, Cephalopoda)
Source: Sci Rep. 2019 May 28;9:7944. doi: 10.1038/s41598-019-44260-w (PMC6538661; doi:10.1038/s41598-019-44260-w)
Supplement: Supplementary file 1 — Supplementary information [file 41598_2019_44260_MOESM1_ESM.pdf]

Supplementary Information for:

Predatory behaviour and taphonomy of a Jurassic belemnoid coleoid (Diplobelida, Cephalopoda)

Dominique Jenny,[1] Dirk Fuchs,[2] Alexander I. Arkhipkin,[3] Rolf B. Hauff,[4] Barbara Fritschi,[5] Christian Klug[1]*

[1]Palaeontological Institute and Museum, University of Zurich, Karl Schmid-Strasse 4, 8006 Zurich, Switzerland.

[2]SNSB-Bayerische Staatssammlung für Paläontologie und Geologie, Richard-Wagner-Str. 10, 80333 Munich, Germany.

[3]Falkland Islands Fisheries Department, FIPASS, Stanley, FIQQ 1ZZ, Falkland Islands.

[4]Urweltmuseum Hauff, Aichelberger Straβe 90, 73271 Holzmaden, Germany.

[5]Bankstrasse 30, 8750 Glarus, Switzerland.

*Correspondence to chklug@pim.uzh.ch

Supplementary Information

Appendix 1: Volume calculations

Head:

1. *Clarkeiteuthis conocauda* (Supplementary figure 6A)

$$cos\alpha = \frac{\frac{r}{2}}{r} \rightarrow \alpha = cos^{-1}\frac{\frac{r}{2}}{r} = \frac{\frac{1.9}{2}}{1.9} = 60° \rightarrow \gamma = 2 \cdot \alpha = 120° = 2.09439rad$$

$$A_{Segment} = \frac{r^2}{2}(\gamma - sin\gamma) = \frac{1.625^2}{2}(2.09439 - sin2.09439) = 1.622cm^2$$

$$A_{Remainder} = r^2\pi - A_{Segment} = 6.67cm^2$$

$$V = A_{Remainder} \cdot h = 6.67cm^2 \cdot 1.5cm = 10.56cm^3$$

2. *Passaloteuthis*

$$cos\alpha = \frac{\frac{r}{2}}{r} \rightarrow \alpha = cos^{-1}\frac{\frac{r}{2}}{r} = \frac{\frac{1.2}{2}}{1.2} = 60° \rightarrow \gamma = 2 \cdot \alpha = 120° = 2.09439rad$$

$$A_{Segment} = \frac{r^2}{2}(\alpha - sin\alpha) = \frac{1.2^2}{2}(2.09439 - sin2.09439) = 0.884cm^2$$

$$A_{Remainder} = r^2\pi - A_{Segment} = 3.64cm^2$$

$$V = A_{Remainder} \cdot h = 3.64cm^2 \cdot 2.2cm = 7.94cm^3$$

3. *Hibolithes semisulcatus*

$$cos\alpha = \frac{\frac{r}{2}}{r} \rightarrow \alpha = cos^{-1}\frac{\frac{r}{2}}{r} = \frac{\frac{3}{2}}{3} = 60° \rightarrow \gamma = 2 \cdot \alpha = 120° = 2.09439rad$$

$$A_{Segment} = \frac{r^2}{2}(\alpha - sin\alpha) = \frac{3^2}{2}(2.09439 - sin2.09439) = 5.528cm^2$$

$$A_{Remainder} = r^2\pi - A_{Segment} = 22.75cm^2$$

$$V = A_{Remainder} \cdot h = 22.75cm^2 \cdot 5cm = 113.73cm^3$$

Body:

(Supplementary figure 6B)

1. *Clarkeiteuthis conocauda*

$$V = \frac{h\pi}{3}(R^2 + Rr + r^2) = \frac{7.75\pi}{3}(1.75^2 + 1.75 * 2.375 + 2.375^2) = 104.364cm^3$$

2. *Passaloteuthis*

$$V = \frac{h\pi}{3}(R^2 + Rr + r^2) = \frac{6.77\pi}{3}(1.2^2 + 1.2 * 1.96 + 1.96^2) = 108.856cm^3$$

3. *Hibolithes semisulcatus*

$$V = \frac{h\pi}{3}(R^2 + Rr + r^2) = \frac{10.5\pi}{3}(3^2 + 3 * 3.5 + 3.5^2) = 349.109cm^3$$

Rostrum solidum:

(Supplementary figure 6C)

1. *Clarkeiteuthis conocauda*

$$V = r^2\pi h\tfrac{1}{3} = 0cm^3$$

2. *Passaloteuthis*

$$V = r^2\pi h\frac{1}{3} = 0.82cm^2 \cdot \pi \cdot 9.82cm \cdot \frac{1}{3} = 6.88\ cm^3$$

3. *Hibolithes semisulcatus*

$$V = r^2\pi h\frac{1}{3} = 0.62cm^2 \cdot \pi \cdot 15.5cm \cdot \frac{1}{3} = 6.24\ cm^3$$

Rostrum cavum/ sheath:

(Supplementary figure 6C)

1. *Clarkeiteuthis conocauda* (Sheath)

$$V = V_1 - V_{phragmocone}$$

$$V_1 = r_1{}^2\pi h\frac{1}{3} = 2.4^2 \cdot \pi \cdot 6.4 \cdot \frac{1}{3} = 38.604cm^3$$

$$V_{phragmocone} = r_2{}^2\pi h\frac{1}{3} = 2.375^2 \cdot \pi \cdot 6.375 \cdot \frac{1}{3} = 37.656cm^3$$

$$V_{Total} = V_1 - V_2 = 0.948cm^3$$

2. *Passaloteuthis* (Rostrum cavum)

$$V = V_1 - V_{phragmocone}$$

$$V_1 = \frac{\pi h}{3} \cdot (R^2 + Rr + r^2) = \frac{\pi 1.75}{3} \cdot (0.82^2 + 0.82 \cdot 1.47 + 1.47^2) = 9.94cm^3$$

$$V_{phragmocone} = r^2\pi h\frac{1}{3} = 1.47^2 \cdot \pi \cdot 1.75 \cdot \frac{1}{3} = 3.97cm^3$$

$$V = V_1 - V_{phragmocone} = 9.94cm^3 - 3.97cm^3 = 5.97cm^3$$

3. *Hibolithes semisulcatus* (Rostrum cavum)

$$V = V_1 - V_{phragmocone}$$

$$V_1 = \frac{\pi h}{3} \cdot (R^2 + Rr + r^2) = \frac{\pi 7.6}{3} \cdot (2^2 + 2 \cdot 0.62 + 0.62^2) = 44.763cm^3$$

$$V_{phragmocone} = r_1{}^2\pi h\frac{1}{3} = 2^2 \cdot \pi \cdot 7.6 \cdot \frac{1}{3} = 31.834cm^3$$

$$V = V_1 - V_{phragmocone} = 44.763cm^3 - 31.834cm^3 = 10.920cm^3$$

Arms:

(Supplementary figure 6D)

1. *Clarkeiteuthis conocauda*

Approximation: $f(x) = \sqrt{ax}$

I. $\sqrt{a \cdot 5.625} = 0.3125$

II. $a \cdot 5.625 = 0.0977$

III. $a = 0.01736$

IV. $f(x) = \sqrt{0.01736 \cdot x}$

$$V_{Onearm} = \pi \int_a^b f(x)^2 dx = \pi \int_0^{5.625} \sqrt{0.01736 \cdot x}^2 dx = 0.863cm^3$$

$$V_{10arms} = 8.63cm^3$$

2. *Passaloteuthis*

Approximation: $f(x) = \sqrt{ax}$

V. $\sqrt{a \cdot 10.76} = 0.38$

VI. $a \cdot 10.76 = 0.14$

VII. $a = 0.01321$

VIII. $f(x) = \sqrt{0.01321x}$

$$V_{Onearm} = \pi \int_a^b f(x)^2 dx = \pi \int_0^{10.76} \sqrt{0.01321x}^2 dx = 2.40cm^3$$

$$V_{10arms} = 24.02cm^3$$

3. *Hibolithes semisulcatus*

Approximation: $f(x) = \sqrt{ax}$

IX. $\sqrt{a \cdot 7} = 0.5$

X. $a \cdot 7 = 0.25$

XI. $a = 0.0357$

XII. $f(x) = \sqrt{0.0357x}$

$$V_{Onearm} = \pi \int_a^b f(x)^2 dx = \pi \int_0^7 \sqrt{0.0357x}^2 dx = 2.75cm^3$$

$$V_{10arms} = 27.5cm^3$$

Proostracum:

(Supplementary figure 6E)

1. *Clarkeiteuthis conocauda*

$$A_{Rectangleminimum} = 2 \cdot r_{minimum} \cdot (h_{total} - r_{minimum}) = 2 \cdot 0.5cm \cdot (3.5cm - 0.5cm) = 3cm^2$$

$$A_{Rectanglemaximum} = 2 \cdot r_{maximum} \cdot (h_{total} - r_{maximum}) = 2 \cdot 1cm \cdot (6cm - 1cm) = 10cm^2$$

$$A_{Semicircleminimum} = \frac{r^2\pi}{2} = \frac{0.5^2\pi}{2} = 0.393cm^2$$

$$A_{Semicirclemaximum} = \frac{r^2\pi}{2} = \frac{1^2\pi}{2} = 1.571cm^2$$

$$V_{minimum} = (A_{Rectangleminimum} + A_{Semicircleminimum}) \cdot 0.01cm = 0.034cm^3$$

$$V_{maximum} = (A_{Rectanglemaximum} + A_{Semicirclemaximum}) \cdot 0.05cm = 0.579cm^3$$

2. *Passaloteuthis*

$$A_{Rectangle} = 2 \cdot r \cdot (h_{total} - r) = 2 \cdot 1.1cm \cdot (6 - 1.1) = 10.71cm^2$$

$$A_{Semicircle} = \frac{r^2\pi}{2} = \frac{1.1^2\pi}{2} = 1.87cm^2$$

$$V_{minimum} = (A_{Rectangle} + A_{Semicircle}) \cdot 0.01cm = 0.126cm^3$$

$$V_{maximum} = (A_{Rectangle} + A_{Semicircle}) \cdot 0.05cm = 0.629cm^3$$

3. *Hibolithes semisulcatus*

$$A_{Rectangle} = 2 \cdot r \cdot (h_{total} - r) = 2 \cdot 2.4cm \cdot (9 - 2.4) = 31.68cm^2$$

$$A_{Semicircle} = \frac{r^2\pi}{2} = \frac{2.4^2\pi}{2} = 9.05cm^2$$

$$V_{minimum} = (A_{Rectangle} + A_{Semicircle}) \cdot 0.01cm = 0.407cm^3$$

$$V_{maximum} = \left(A_{Rectangle} + A_{Semicircle}\right) \cdot 0.05cm = 2.036cm^3$$

Phragmocone

(Supplementary figure 6G)

1. *Clarkeiteuthis conocauda*

$$V_1 = {r_1}^2 \pi h \frac{1}{3} = 2.375^2 \cdot \pi \cdot 6.375 \cdot \frac{1}{3} = 37.66cm^3$$

$$V_2 = {r_2}^2 \pi h \frac{1}{3} = 2.370^2 \cdot \pi \cdot 6.370 \cdot \frac{1}{3} = 37.47cm^3$$

$$V_{Total} = V_1 - V_2 = 0.188cm^3$$

2. *Passaloteuthis*

$$V_1 = {r_1}^2 \pi h \frac{1}{3} = 1.8^2 \cdot \pi \cdot 6.3 \cdot \frac{1}{3} = 21.38cm^3$$

$$V_2 = {r_2}^2 \pi h \frac{1}{3} = 1.795^2 \cdot \pi \cdot 6.295 \cdot \frac{1}{3} = 21.24cm^3$$

$$V_{Total} = V_1 - V_2 = 0.136cm^3$$

3. *Hibolithes semisulcatus*

$$V_1 = {r_1}^2 \pi h \frac{1}{3} = 2^2 \cdot \pi \cdot 7.6 \cdot \frac{1}{3} = 31.83cm^3$$

$$V_2 = {r_2}^2 \pi h \frac{1}{3} = 1.995^2 \cdot \pi \cdot 7.595 \cdot \frac{1}{3} = 31.66cm^3$$

$$V_{Total} = V_1 - V_2 = 0.179cm^3$$

Phragmocone sections

(Supplementary figure 6)

Approximation: $f(x) = \sqrt{ax}$

I. $\sqrt{a \cdot \frac{r}{5}} = r$

II. $\frac{ar}{5} = r^2$

III. $\frac{a}{5} = r$

IV. $a = 5r$

V. $f(x) = \sqrt{5r \cdot x}$

$$V = A \cdot h$$

$$A = 2\pi \int_0^{\frac{r}{5}} f(x) \cdot \sqrt{1 + f'(x)^2} dx = 2\pi \int_0^{\frac{r}{5}} \sqrt{5r \cdot x} * \sqrt{1 + \frac{5r^2}{4x}}\, dx$$

Clarkeiteuthis conocauda total volume: 0.696 cm^3 (see Supplementary table 1).

Passaloteuthis total colume: 0.507 cm^3 (see Supplementary table 2).

Hibolithes semisulcatus total volume: 0.3886 cm^3 (see Supplementary table 3).

Supplementary Reference

84. Fuchs, D. Fossil erhaltungsfähige Merkmalskomplexe der Coleoidea (Cephalopoda) und ihre phylogenetische Bedeutung. *Berliner Paläobiol. Abh*. **8**, 1–122 (2006).

Supplementary Tables

Septum	Radius (cm)	Height (cm)	A (cm^2)	Volume (cm^3)
1	2.313	0.005	25.985	0.130
2	2.188	0.005	22.636	0.113
3	2.000	0.005	13.056	0.065
4	1.875	0.005	15.442	0.077
5	1.813	0.005	14.197	0.071
6	1.625	0.005	10.831	0.054
7	1.563	0.005	9.829	0.049
8	1.438	0.005	7.996	0.040
9	1.250	0.005	5.660	0.028
10	1.125	0.005	4.364	0.022
11	0.938	0.005	2.785	0.014
12	0.750	0.005	1.609	0.008
13	0.650	0.005	1.134	0.006
14	0.600	0.005	0.932	0.005
15	0.550	0.005	0.754	0.004
16	0.500	0.005	0.598	0.003
17	0.450	0.005	0.463	0.002
18	0.400	0.005	0.348	0.002
19	0.350	0.005	0.252	0.001
20	0.300	0.005	0.174	0.001
21	0.250	0.005	0.113	0.001
22	0.200	0.005	0.066	0.000

Supplementary table 1. Volume calculations for the septa of *Clarkeiteuthis conocauda.*

Septum	Radius (cm)	Height (cm)	A (cm^2)	Volume (cm^3)
1	1.8	0.005	13.955	0.069775
2	1.7	0.005	12.1121	0.0605605
3	1.6	0.005	10.4233	0.0521165
4	1.56	0.005	9.78989	0.0489495
5	1.52	0.005	9.18019	0.045901
6	1.4	0.005	7.49004	0.0374502
7	1.32	0.005	6.47594	0.0323797
8	1.28	0.005	6.00173	0.0300087
9	1.24	0.005	5.54899	0.027745
10	1.2	0.005	5.11737	0.0255869
11	1.1	0.005	4.12851	0.0206426
12	1	0.005	3.26409	0.0163205
13	0.9	0.005	2.51841	0.0125921
14	0.8	0.005	1.88548	0.0094274
15	0.7	0.005	1.35898	0.0067949
16	0.6	0.005	0.932085	0.0046604
17	0.5	0.005	0.597666	0.0029883
18	0.4	0.005	0.347844	0.0017392
19	0.3	0.005	0.17397	0.0008699
20	0.2	0.005	0.066265	0.0003313

Supplementary table 2. Volume calculations for the septa of *Hibolithes semisulcatus*.

Septum	Radius (cm)	Height (cm)	A (cm^2)	Volume (cm^3)
1	2.000	0.005	13.056	0.065
2	1.800	0.005	13.955	0.070
3	1.700	0.005	12.112	0.061
4	1.500	0.005	8.884	0.044
5	1.350	0.005	6.846	0.034
6	1.300	0.005	6.236	0.031
7	1.200	0.005	5.117	0.026
8	1.000	0.005	3.264	0.016
9	0.800	0.005	1.886	0.009
10	0.740	0.005	1.557	0.008
11	0.680	0.005	1.266	0.006
12	0.620	0.005	1.010	0.005
13	0.560	0.005	0.788	0.004
14	0.500	0.005	0.598	0.003
15	0.440	0.005	0.438	0.002
16	0.380	0.005	0.307	0.002
17	0.320	0.005	0.203	0.001
18	0.260	0.005	0.124	0.001
19	0.200	0.005	0.066	0.000

Supplementary table 3. Volume calculations for the septa of *Hibolithes semisulcatus*.

Supplementary Figures

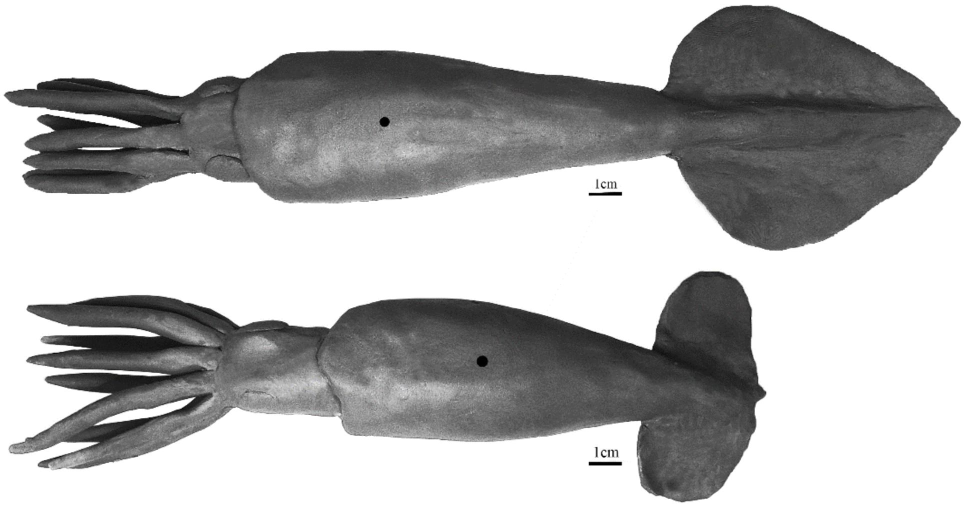

Supplementary figure 1: Top: Clay model of *Hibolithes semisculatus*, half natural size. Bottom: life-sized reconstruction of *Clarkeiteuthis conocauda.* Both made from clay. The black dot marks the center of buoyancy in each specimen. Fin sizes and shapes are hypothetical.

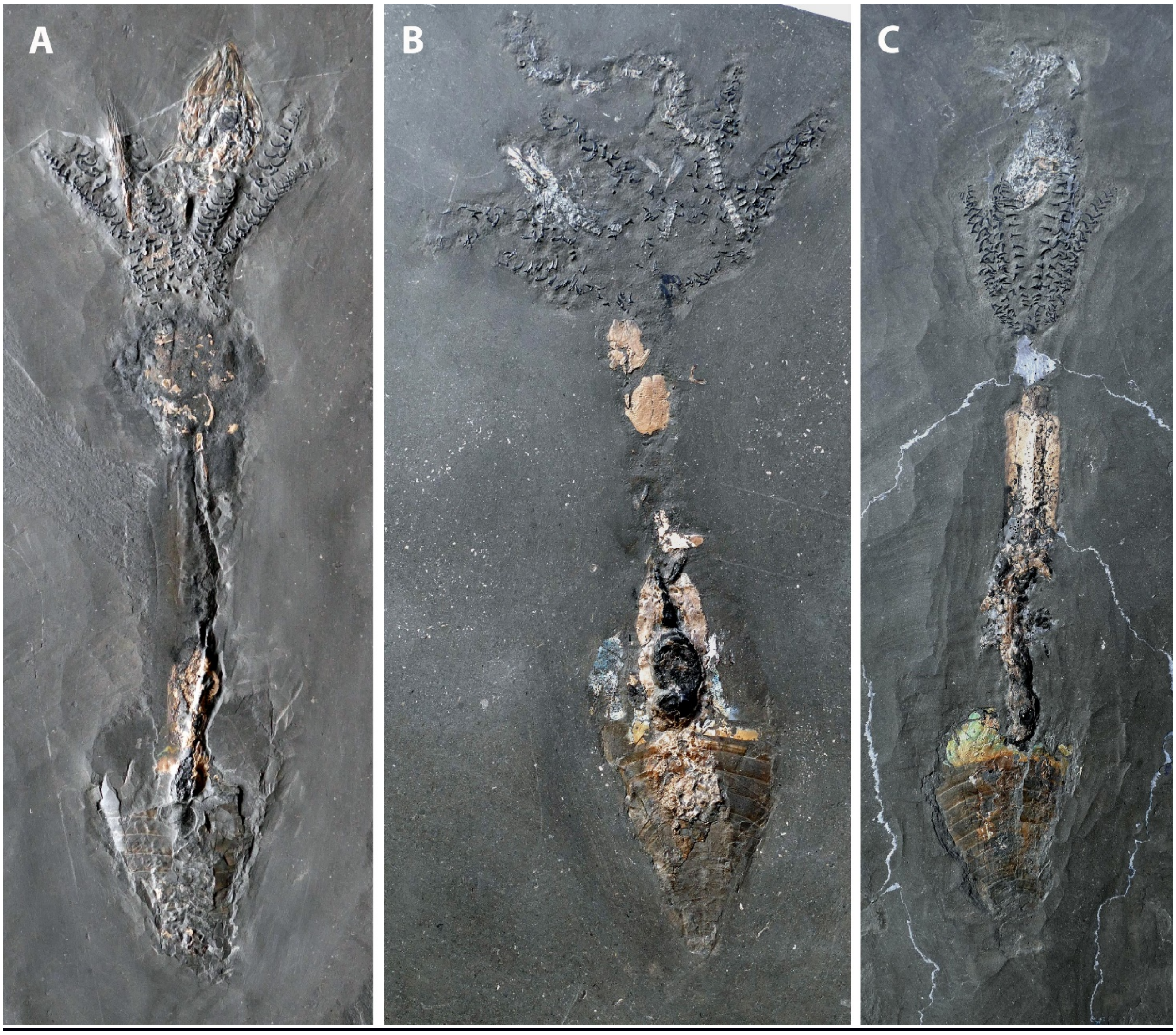

Supplementary figure 2: Additional specimens of *Clarkeiteuthis conocauda* with *Leptolepis bronni* in its arm crown; Posidonienschiefer, Toarcian, Early Jurassic of Holzmaden (Germany); all photos courtesy G. Schweigert (Stuttgart). A, specimen figured by J. Klaschka[10]. B, C, two specimens from the collection of G. Weber (Rechberghausen), both from the Koblenzer beds. B, note the fractured vertebral column and the head of the comparatively large fish pointing out of the arm crown; arms are spread. C, in this specimen, the rather small fish lies with the head facing the buccal mass; the arms are well preserved and contracted. Also note the partially phosphatized proostracum.

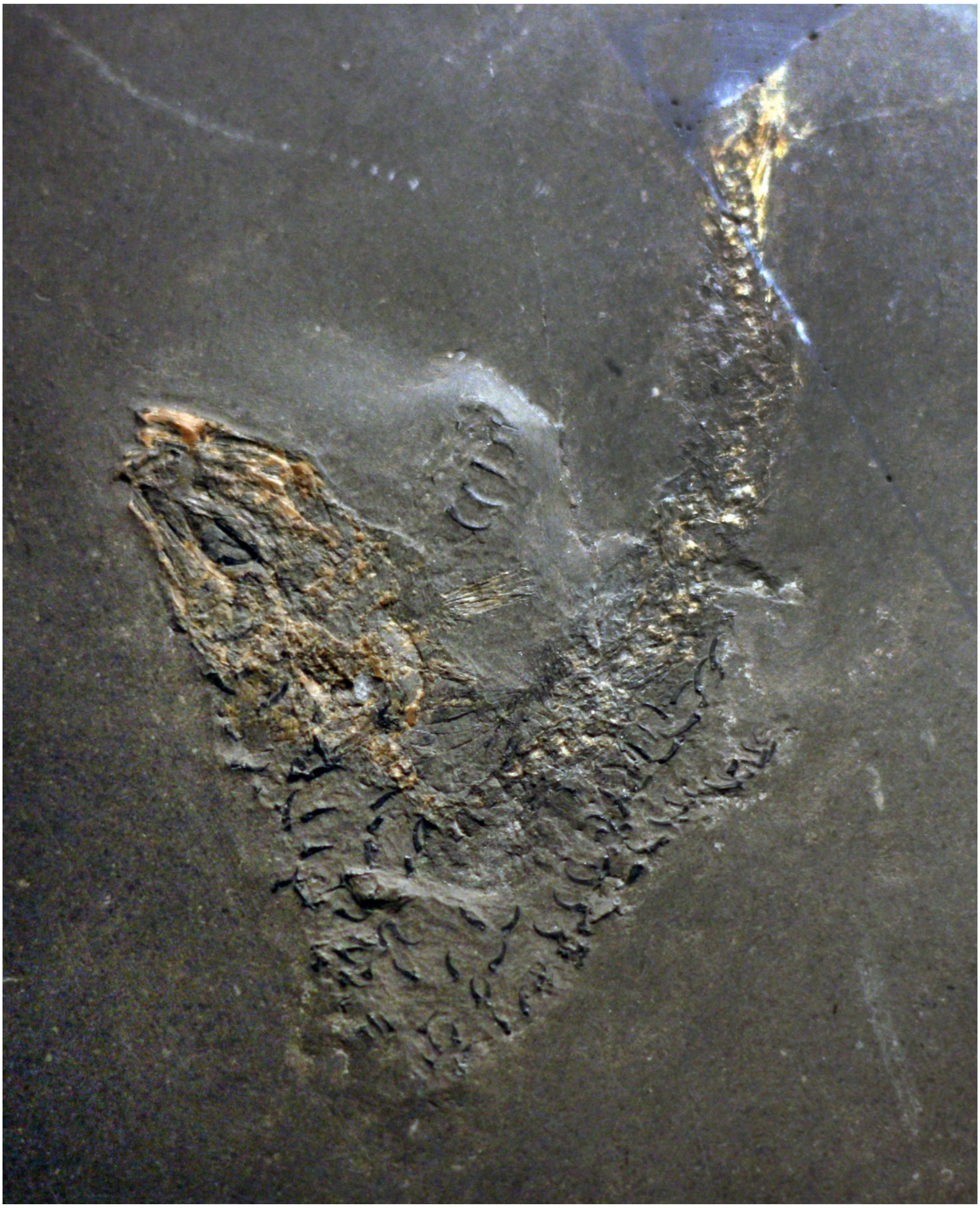

Supplementary figure 3. Counterpart of the main specimen of *Clarkeiteuthis conocauda* with *Leptolepis bronni* in its arm crown; Posidonienschiefer, Toarcian, Early Jurassic of Holzmaden (Germany). In this case, only the arm crown with the fish is preserved.

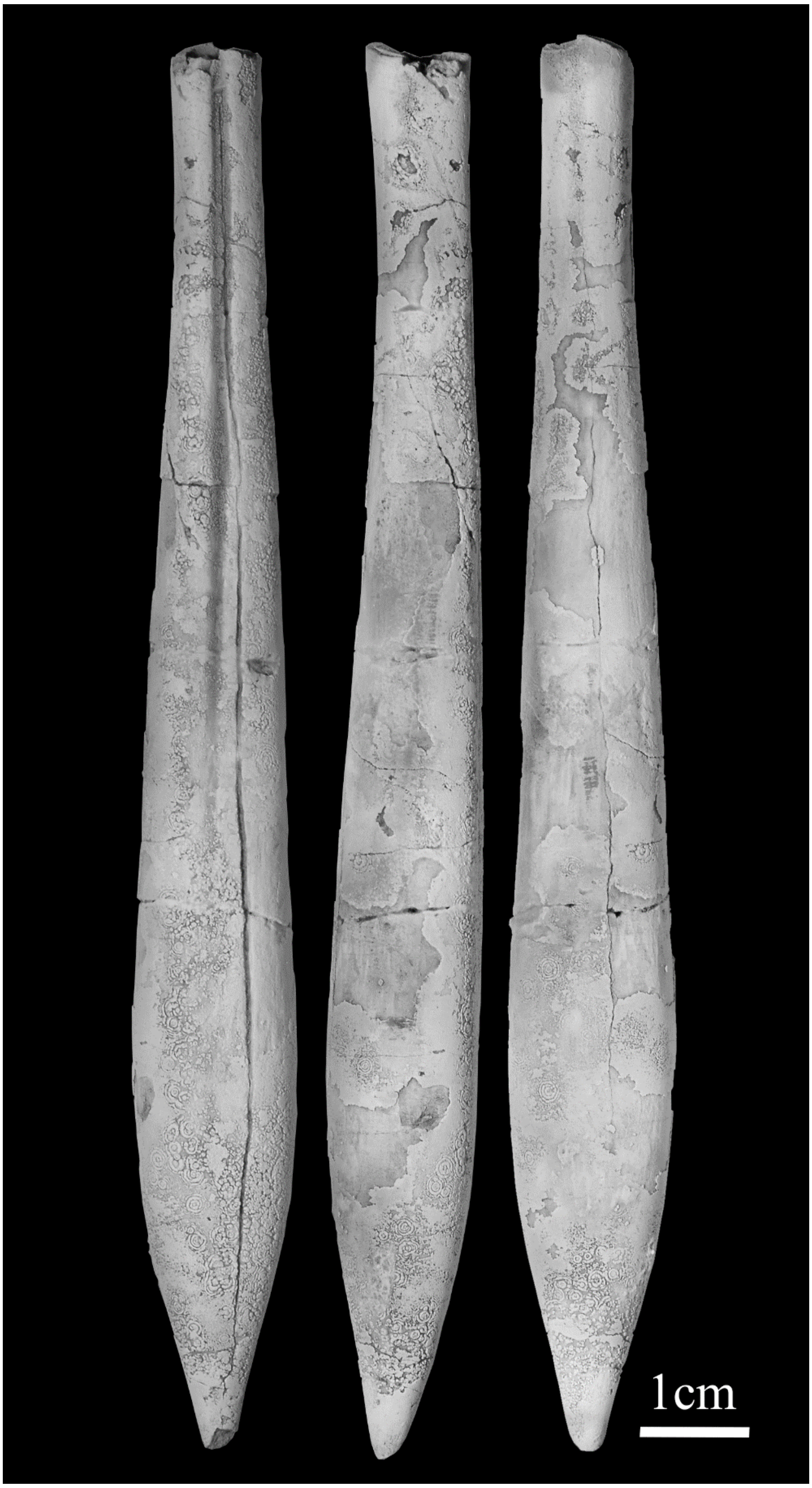

Supplementary figure 4: *Hibolithes* sp. indet., PIMUZ 24398; Herznach Formation, Callovian, Liesberg (Basel-Land, Switzerland). The specimen was whitened with NH_4Cl. 1. ventral, 2. lateral, 3. dorsal view the rostrum solidum.

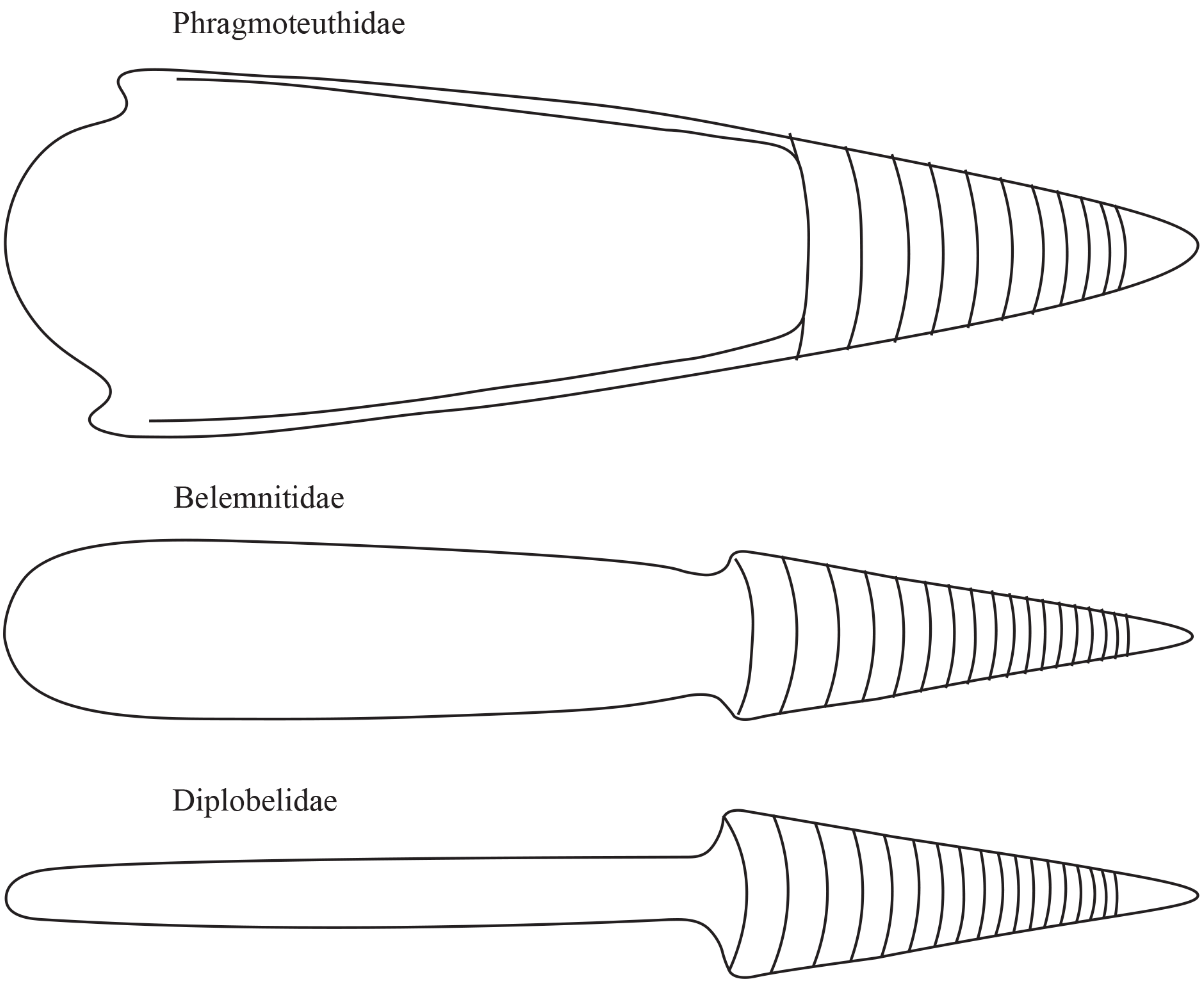

Supplementary figure 5: Reconstructions of the hard parts (phragmocone and proostracum; without rostrum) of the Phragmoteuthida, Belemnitida and Diplobelida[83,84].

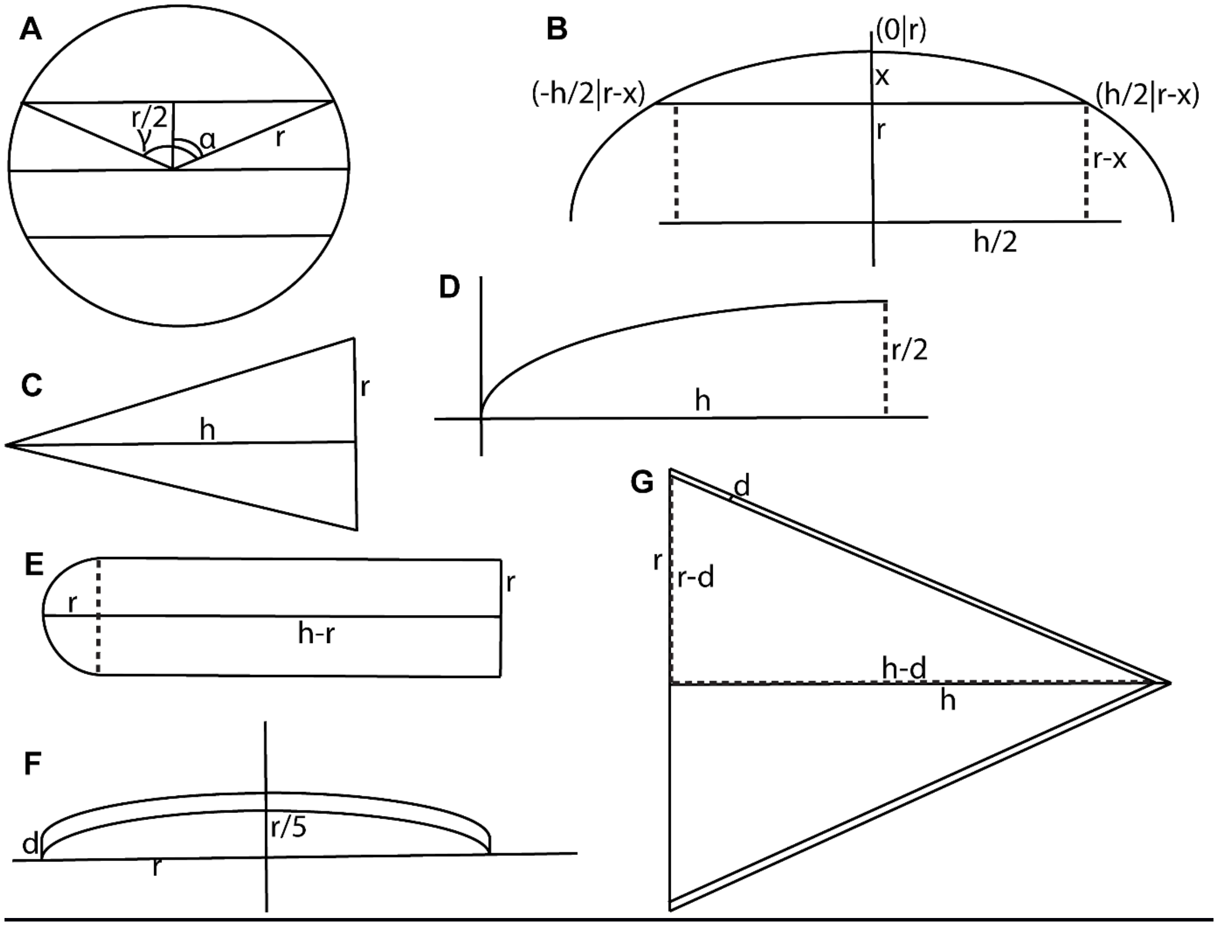

Supplementary figure 6: Graphs showing details of the geometric forms used for the volume calculations. A, graph of the head cross section. B, Graph of the body (longitudinal). C, Graph of the rostrum. D, Graph of an arm. E, Graph of the proostracum. F, *Graph of* one *septum* (cross section). G, graph of the whole phragmocone (without septa).
